# Clinicopathological and Sero-Molecular Detection of *Mycoplasma capricolum* subsp. *capripneumoniae* in Goats in Southern Areas of Pakistan

**DOI:** 10.1155/2022/9508810

**Published:** 2022-10-03

**Authors:** Faiz Ur Rehman, Farhan Anwar Khan, Muhammad Saeed, Mehboob Ali, Hayatullah Khan, Faisal Ahmad, Qudrat Ullah

**Affiliations:** ^1^College of Veterinary Sciences, Faculty of Animal Husbandry and Veterinary Sciences, The University of Agriculture, Peshawar, Pakistan; ^2^Livestock and Dairy Development (Research), Peshawar, Khyber Pakhtunkhwa, Pakistan; ^3^Livestock and Dairy Development (Extension), Peshawar, Khyber Pakhtunkhwa, Pakistan

## Abstract

Contagious caprine pleuropneumonia (CCPP) is a highly fatal infectious disease of goats, caused by *Mycoplasma capricolum* subsp. *capripneumoniae* (Mccp). This disease is causing huge economic losses to the goat industry in Pakistan. However, little is known about the epidemiology of CCPP, especially in the hard areas of Khyber Pakhtunkhwa (KP), Pakistan, despite having a huge population of goats. Therefore, this study aimed to elucidate sero-molecular epidemiology and pathology associated with Mccp infection in goats in southern areas of KP including Dera Ismail Khan (DI Khan), Bannu, Karak, and Kohat. A total of 200 (50 from each area) serum samples were collected from clinically infected goats, whereas 600 various samples (nasal swab *n* = 50, pleural fluid *n* = 50, lungs *n* = 50 at each selected area of study) were collected from live goats showing respiratory clinical signs and dead/slaughter goats having lesions in the lungs/pleura. A commercial competitive ELISA kit confirmed anti-Mccp antibodies in altogether 17% of serum samples, while area-wise seroprevalence was recorded as follows: Kohat, 28%, Bannu, 18%, DI Khan, 14%, and Karak, 8%. Moreover, a total of 5.5% of samples collected from clinically positive live and dead goats for Mccp were found by species-specific PCR, whereas area-wise molecular prevalence of Mccp was found in 3% samples from Kohat, 7.33%, Bannu, 6%, Khan, 5.33%, and Karak, 3.33%. Of 400 clinically examined goats, 242 (60%) had nasal discharge, 207 (51%) had pyrexia, 50.75% (203) had coughing, 48.25% (193) had pneumonia, 23% (92) had lacrimation, 7.75% (31) had pneumonia with lacrimation, and 10 (2.5%) showed all signs. Of the total 200 dead/slaughtered goats, pleural fluid was found in 36 goats and consolidation and red hepatization were observed in 40 and 42 goats, respectively. The present study found the presence of prevailing Mccp strain in the goat population of the study area. The highest prevalence of Mccp was found in collected samples from Kohat by PCR. The highest seroprevalence of Mccp was found in serum samples collected from Kohat by ELISA.

## 1. Introduction

In Pakistan, the livestock sector is dominated by the largest population of goats (Pakistan Economic Survey 2020-2021). Therefore, the goat is known as “poor's man cow” in the subcontinent [1]. The most important threats to livestock population, especially goats, are respiratory diseases worldwide. Among respiratory infections, *Mycoplasma*-linked infections are responsible for massive economic losses in small ruminants in developing countries [2]. Mycoplasmas are the simplest self-replicating microorganisms, lacking a cell wall; however, they are highly species-specific and successful pathogens [3–5]. *Mycoplasma*-related infections are widely distributed all over the world, almost in all developing countries of Middle East Asia, South East Asia, and Africa [6, 7].

The most dreadful respiratory mycoplasmosis in goats is contagious caprine pleuropneumonia (CCPP). CCPP was first clinically reported in 1873 in Algeria [8]. Then, in 1881, CCPP was proved as a contagious infection in goats [9]. After a century, in 1976, the actual causative agent of CCPP, Mccp, was first isolated and characterized [10–12]. Mccp has been isolated from 13 countries but reported in 40 countries so far [13]. CCPP caused by Mccp is responsible for 100% morbidity and 60–80% mortality in goat flocks [14]. Mccp belongs to the *Mycoplasma mycoides* cluster. There are six species and subspecies in the *Mycoplasma mycoides* cluster [15], which causes disease in small ruminants as well as large ruminants. It shares multiple genomic properties or multiple phenotypic properties [16]. *Mycoplasma mycoides* cluster is further divided into two subgroups: *Mycoides* and *Capricolum*. *Mycoides* are further divided into three subspecies; *Mycoplasma mycoides* subsp*. mycoides* small colony (MmmSC), *Mycoplasma mycoides* subsp*. mycoides* large colony (MmmLC), and *Mycoplasma mycoides capri* (Mmc). *Capricolum* includes three subspecies: *Mycoplasma capricolum* subsp. *capricolum* (Mcc), *Mycoplasma capricolum* subsp. *capripneumoniae* (Mccp), and *Mycoplasma* subsp. *bovine* 7^th^ group (BG7) [12, 17]. The non-*Mycoplasma* cluster subspecies are *Mycoplasma ovipneumoniae, Mycoplasma putrefaciens*, and *Mycoplasma agalactiae* [7, 18].

*Mycoplasma capricolum* subsp. *capripneumoniae* is a very fastidious slow-growing *Mycoplasma* with incubation time ranging from 7 to 10 days. The incubation may vary between 5 and 28 days. The first clinical sign of Mccp infection in goats is high body temperature (41°C) and reluctance to walk but the animal continues to feed intake. Then, the respiratory signs appear prominently with painful and deep respiration and frequent coughing. In the advanced stages, the animals are reluctant to move, continue salivation, and exhibit mucopurulent nasal discharge. In some cases, the animals have marked lameness, diarrhea, and nervous signs [19]. The gross pathological lesions associated with Mccp infection are restricted to the pleura and lung. The lungs are usually infected unilaterally, but bilateral infection has also been reported with CCPP [20]. There is massive red hepatization and pleurisy with fibrinous pleuropneumonia and straw-color pleural fluid. The necrotic areas on the lungs are sequestered and of black discoloration [19]. Histopathological lesions associated with Mccp infections are pulmonary emphysema, condensing of interlobular septa almost in all cases, and atelectasis [19, 21].

The identification of Mccp infection is difficult on the basis of clinical signs and symptoms because there is variation in clinical signs and symptoms [22]. Serological tests are most commonly used for the diagnosis of mycoplasmas. The common serological tests are growth inhibition test (GIT), indirect or passive haemagglutination assay (IHA/PHA), enzyme-linked immunosorbent assay (ELISA), complement fixation test (CFT), latex agglutination tests (LAT), and fluorescent antibodies test (FAT) [23–26].

Although the PCR is the most sensitive and accurate technique for the diagnosis of Mccp, it is a time-consuming, expensive technique and hardly detects Mccp in treated animals with antimicrobials. On the other hand, ELISA could diagnose the Mccp infection in treated animals as well as recovered animals. Therefore, we used a combination of serological, molecular, and pathological techniques for the detection of actual prevalence of Mccp local strain in goats in study areas.

## 2. Materials and Methods

### 2.1. Study Area

The current study was conducted in southern areas of Khyber Pakhtunkhwa including Khan, Bannu, Karak, and Kohat. These areas were visited for collection of samples from goats clinically suspected of CCPP.

### 2.2. Collection of Samples

Samples were collected from the suspected goat population in districts of Khan, Bannu, Karak, and Kohat of Khyber Pakhtunkhwa. A total of 800 samples were collected from clinically suspected and dead or slaughtered goats for CCPP. A total of 200 (50 from each area) serum samples were collected from clinically infected goats, whereas 600 various samples (nasal swab *n* = 50, pleural fluid *n* = 50, lungs *n* = 50 at each selected area of the study) were collected from live goats showing respiratory clinical signs and dead/slaughter goats having lesions in the lungs/pleura. Blood samples were collected from the jugular vein for serodiagnosis of Mccp infection, whereas nasal swabs, lung tissue, and pleural fluid were collected from CCPP suspected goats for molecular detection of Mccp by PCR. Pleural fluid was collected in a sterile tube. Sterile cotton nasal swabs were inserted deep into the nasal passage to get the secretions of the goat. The lung tissue was collected in sterile sealable plastic bags and transported in an ice box to Pathology Laboratory, College of Veterinary Sciences, the University of Agriculture, Peshawar, and stored at −20°C or −86°C freezer until used. For histopathology, lung tissues were collected and transported in 10% buffered formalin to the laboratory.

### 2.3. Sero-Epidemiological Analysis of Mccp Infection by ELISA

The serum samples were tested with a commercially available competitive ELISA kit (IDEXX-USA). The manufacturer's protocol was followed for cELISA.

### 2.4. Molecular Detection of Mccp by PCR

#### 2.4.1. Genomic DNA Extraction and Quantification

Genomic DNA (gDNA) was extracted using GeneJET Genomic DNA Purification Kit made by Thermo Scientific, USA. The DNA extraction procedure was followed according to manufacturer instructions. The extracted gDNA was quantified with Nanodrop (Thermofisher-Finland). The DNA was diluted according to the desired level for PCR as reported elsewhere [27, 28].

#### 2.4.2. Selection of Primers for PCR

The following set of primers was used for the detection of Mccp as reported previously [28–30]. Specific primers of *Mycoplasma capricolum* subsp*. capripneumoniae* were used for targeting a specific Mccp gene and generating an amplicon of 316 bp:

  Mccp.spe-F: 5′-ATC ATT TTT AAT CCC TTC AAG-3′
  Mccp.spe-R: 5′-TAC TAT GAG TAA TTA TAA TAT ATG CAA-3′

#### 2.4.3. Preparation of PCR and Conditions

PCR was performed using a PCR Thermal Cycler (Bio-Rad T100 USA). A total of 25 *µ*l PCR reaction was prepared consisting of 1.75 *µ*l forward primer and 1.75 *µ*l reverse primer, 8 *µ*l nuclease-free water, 10 *µ*l PCR Master Mix, and 3.5 *µ*l DNA template [28, 31]. Initial denaturation of template DNA was done at 94°C for 5 min followed by cycles of denaturation at 94°C for 30 sec, annealing at 53°C for 60 sec, and extension at 72°C for 90 sec and final extension was performed at 72°C for 5 min.

#### 2.4.4. Gel Electrophoresis

PCR product was run on 1.5% agarose gel. After loading 6 *µ*l of PCR products, including samples, positive control, negative control, and DNA ladder of 1 kb or 100 bp, agarose gel was run on the gel for 35 min at 120 V. The PCR products were visualized by the gel documentation system (FastGene, Germany).

### 2.5. Histopathological Study

Tissue samples (Trachea and lungs) were collected and preserved in 10% buffered formalin. Tissues were processed according to the standard protocol as adapted in [32, 33].

### 2.6. Statistical Analysis

All the data was collected and arranged in a Microsoft Excel worksheet. The collected data were then subjected to Statistical Package SPSS v20. A Chi-Square test was performed for analyzing the data.

## 3. Results

### 3.1. Seroprevalance of Mccp in Goats by ELISA

**Figure 1:**
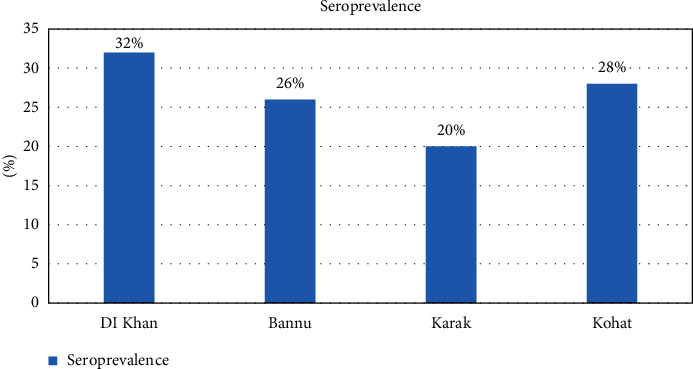
Graphical representation of seroprevalence on cELISA in southern areas of KPK.

The ELISA was performed on 200 serum samples from goats for the detection of anti-Mccp antibodies. From Dera Ismail Khan, of 50 serum samples, 7 (14%) were positive for Mccp, whereas 18% of samples were positive from Bannu. Samples taken from Karak showed 8% positivity for Mccp. Interestingly, the highest number of samples (28%) positive for Mccp was found in the Kohat district. The overall seroprevalence of Mccp was found at 17% in southern areas of KP Pakistan. Statistical analysis by *χ*2 showed a significant association (*P* > 0.05) among four districts (Tables 1-2, Figure 1).

### 3.2. Molecular Prevalence of Mccp in Goats in Southern Areas of KP by PCR

**Figure 2:**
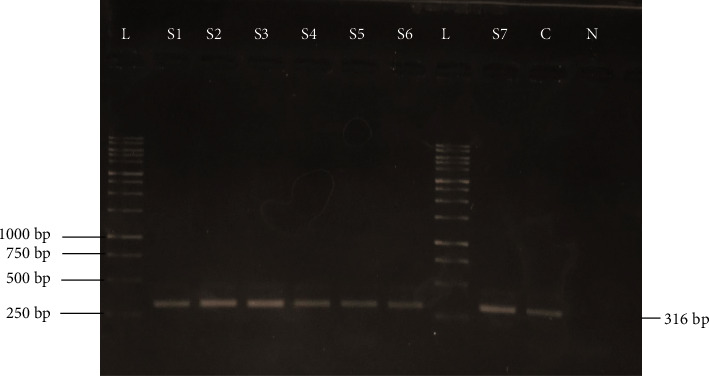
PCR result of *Mycoplasma capricolum* subsp. *capripneunomiae* with an amplicon size of 316 in samples collected from goats. L = 1 Kb DNA ladder, samples = S1, S2, S3, S4, S5, S6, and S7, C = positive control, and N  = negative control.

**Figure 3:**
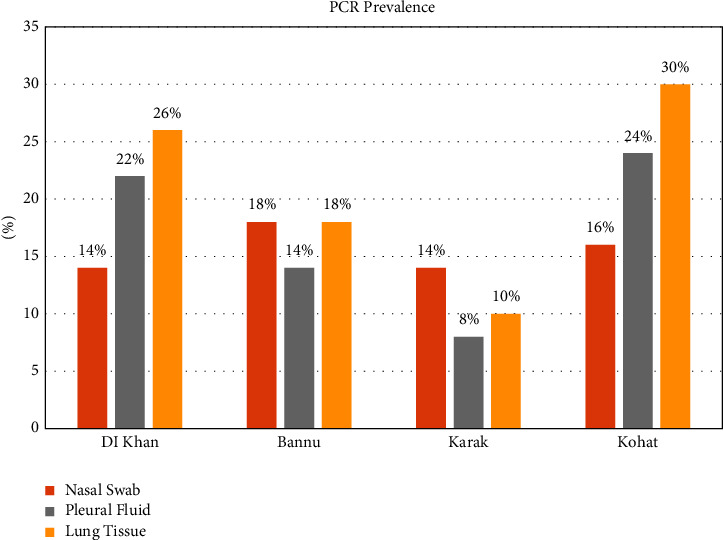
Graphical representation of sample-wise prevalence *Mycoplasma capricolum* subsp. *capripneumoniae* by PCR in southern areas of KPK.

**Figure 4:**
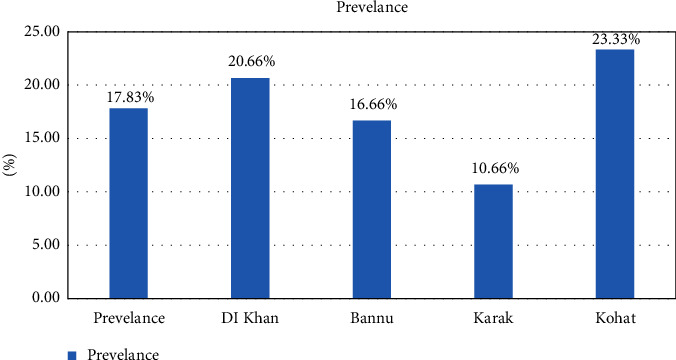
Graphical representation of total prevalence of *Mycoplasma capricolum* subsp. *capripneumoniae* by PCR in southern areas of KPK.

For the molecular prevalence of Mccp, PCR was performed on a total of 600 samples from southern areas of KPK, including 150 samples (50 nasal swabs, 50 pleural fluids, and 50 lung tissues) collected from each area, respectively (DI Khan, Bannu, Karak, and Kohat). The causative agent of CCPP was detected in 5% of samples from DI Khan, whereas 6% and 3% of samples were found positive for Mccp from Bannu and Karak, respectively. The molecular tool also detected the highest number (7%) of positive samples from the Kohat district. However, an overall molecular prevalence of Mccp was found at 6% in southern areas of KP (Tables 3-4, Figures 2–4).

### 3.3. Clinicopathological Study of *Mycoplasma capricolum* subsp. c*apripneumoniae*

#### 3.3.1. Clinical Manifestation of Mccp Infection in Goats

**Figure 5:**
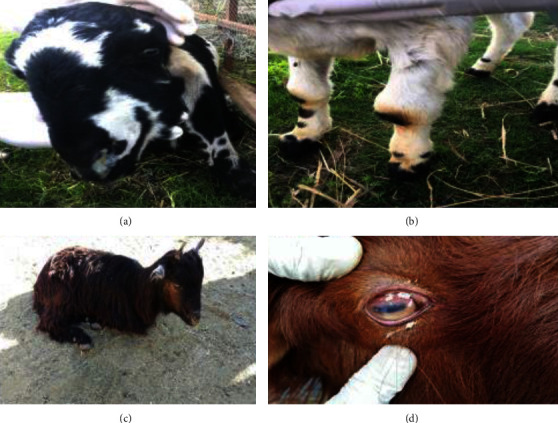
(a) Nasal discharge and lacrimation. (b) Synovial joint swelling. (c) Nasal discharge, lacrimation, and fever. (d) Conjunctivitis.

A total of 400 goats (100 in each district) were examined for clinical signs of CCPP including body temperature, coughing, nasal discharge, lacrimation, conjunctivitis, and arthritis. In a total of 400 goats, 242 (60.5%) animals showed nasal discharge, 51% exhibited pyrexia (103–104°F), 51% showed cough, and 48% showed signs of pneumonia. Conjunctivitis was recorded in 24% of animals, while lacrimation and arthritis were recorded in 23% and 3% animals, respectively. Only 8% of animals showed both pneumonia and lacrimation concomitantly, whereas 3% of animals showed all clinical signs mentioned above (Table 5, Figure 5).

#### 3.3.2. Gross Pathology

**Figure 6:**
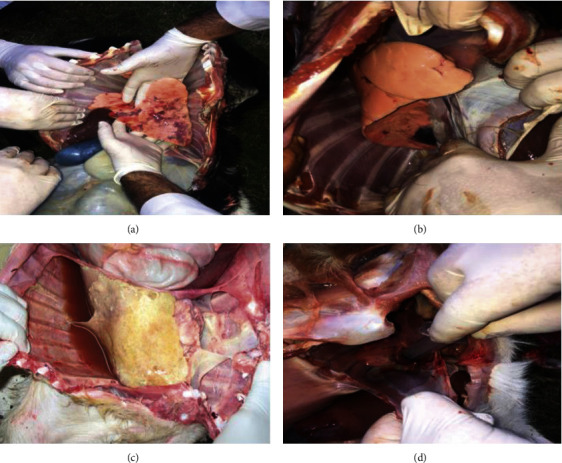
(a) Gross lesion in the lungs of a goat at post mortem examination suffering from respiratory symptoms suspected for Mccp. The lungs showing red hepatization, accumulation of pleural fluid, and haemorrhages (b). Gross lesion in lungs of a goat at post mortem examination suffering from the respiratory symptom suspected for Mccp. The lungs showing consolidation and accumulation of pleural fluid (c). Gross lesion in the lungs of a goat at post mortem examination suffering from the respiratory symptom suspected for Mccp. Accumulation of pleural fluid in pleural cavity (d). Gross lesion in the lungs of a goat at post mortem examination suffering from the respiratory symptom suspected for Mccp. The lungs showing consolidation and accumulation of pleural fluid collected with a sterile syringe.

Pathological investigations (gross and histopathological) were carried out on tissue samples from a total of 200 necropsied/slaughtered animals. The tissue samples were collected from different slaughterhouses. A total of 50 tissue samples were collected from each area (Dera Ismail Khan, Bannu, Karak, and Kohat). Lung tissue samples from animals (*n* = 26) at DI Khan were grossly normal, whereas samples from animals (*n* = 24) showed various gross lesions including consolidation of the affected lungs, red hepatization (unilateral), and adhesion of the lungs with the thoracic cavity. Tissue samples from animals (*n* = 34) at Bannu were grossly normal, whereas samples from 16 animals were infected showing pleural effusion (*n* = 9), red hepatization (*n* = 7), and adhesion of the lungs with the thoracic cavity (*n* = 9) from infected animals. From Karak, tissue samples from animals (*n* = 41) were grossly normal, while 9 animals who were grossly infected exhibited different lesions that includes pleural fluid (*n* = 4), red hepatization (*n* = 5), and adhesion of the lungs (*n* = 5). From Kohat, samples from 23 animals were normal, while 27 had gross lesions (Table 6, Figure 6).

#### 3.3.3. Histopathology

**Figure 7:**
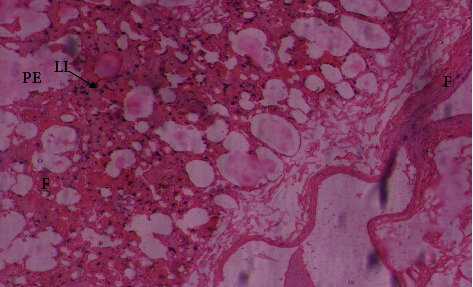
The lungs of goats suffering from respiratory syndrome showing fibrosis (F), pulmonary emphysema (PE), and infiltartion of leucocytes (IL) suffering from Mccp (H & E stain).

**Figure 8:**
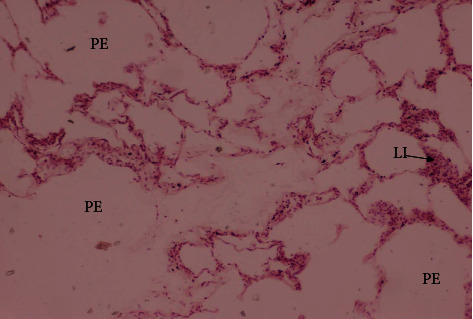
Lungs of goats suffering from respiratory syndrome showing pulmonary emphysema (PE) and leukocytic infiltartion (LI) suffering from Mccp (H & E stain).

From the total lung tissue samples collected from animals (*n* = 200), samples (*n* = 50) were collected from each area (Dera Ismail Khan, Bannu, Karak, and Kohat). From Khan, of 50 animal samples, only 12 animal tissue samples exhibited histopathological lesions including pulmonary emphysema, leucocyte infiltration, atelectasis, and thickening of interalveolar septa. Histopathological lesions have been observed in tissue samples from only six animals of 50 animals from Bannu, while samples from four animals of Karak exhibited similar lesions. From Kohat, 11 animal tissue samples were found to have pulmonary emphysema, leucocyte infiltration, atelectasis, and thickening of interalveolar septa (Table 7, Figures 7and 8).

## 4. Discussion

Mycoplasmosis causes serious threat and massive economic losses in small ruminants (sheep and goats) in developing countries [2, 34, 35]. Mycoplasmosis causes high morbidity and mortality [2]. Mycoplasmosis is a pathogenic bacteria for multisystems and is collectively caused by *Mycoplasma mycoides* cluster. Mycoplasma is highly prevalent all over the world almost in all developing countries of Middle East Asia, South East Asia, and Africa [6, 7]. Mycoplasma is the smallest and slow-growing bacteria. It can cause disease in different species of animals and also cause disease in humans. Mycoplasma causes respiratory disorders, genital disorders, eye lesions, arthritis, and mastitis [36, 37]. Mycoplasmosis in goats is known as CCPP. The targeted area for Mccp in the host is the respiratory system and is restricted to the thoracic cavity [15].

The species-specific primers have enabled an advanced technique to be applied directly to the clinical samples, i.e., nasal swabs, fluid samples, and tissue samples [38, 39]. A total of 600 different samples were collected from naturally dead and slaughtered goats from different study areas. Those samples were subjected to PCR for the identification of *Mycoplasma capricolum* subsp. *capripneumoniae*. Of 600, only 33 samples were from nasal discharges, pleural fluids, and lung tissues. 10 (5%), 15 (7.5%), and 8 (4%) were detected with *Mycoplasma capricolum* subsp. *capripneumoniae*. Of 150 samples, 8 (5.33%) from Khan, 9 (6%) from Bannu, 5 (3.33%) from Karak, and 11 (7.33%) from Kohat were positive from nasal discharges, pleural fluid, and lung tissues on PCR analysis. The total positive percentage of *Mycoplasma capricolum* subsp. *capripneumoniae* on PCR was 5.5%. Similar studies were also conducted in [40–43]. The remaining samples were found negative on PCR. That might be due to other *Mycoplasma* cluster species that cause diseases in goats and them presenting similar clinical signs.

The cELISA results showed 17% overall seroprevalence at the southern areas of KP. Of 50 serum samples, 7 (14%) from DI Khan, 9 (18%) from Bannu, 4 (8%) from Karak, and 14 (28%) from Kohat were positive. The remaining 166 serum samples were negative on cELISA. The same study was reported in [34, 44, 45] in the northern areas (Swat and Buner) of KPK, Pakistan. But the results were in contrast because samples were collected randomly from goats, while in the current study, the serum samples were collected from suspected goats. It means that the disease is prevalent throughout the country. Area-wise distribution of the Mccp was more prevalent in Kohat as compared to Khan, Bannu, and Karak. The statement is justified by the inhabitant of the small ruminants of former nomads, constantly moving from place to place in search of pastures. The reason contributed to stress, which is predisposed to Mccp infection in goats. And these nomads cross the border easily and enter the area which leads to transboundary transmission of the disease; the same observation was also reported by the finding in [42].

The third objective of the work was the clinicopathological study of Mccp in southern areas of Khyber Pakhtunkhwa Pakistan. In the present study, a total of 400 goats were examined for clinical signs and symptoms. The respiratory signs were common features for infected goats followed by pyrexia found in 207 (51%) goats, coughing in 203 (50.75%), pneumonia in 193 (48.25%), nasal discharge in 242 (60.5%), lacrimation in 92 (23%), arthritis in 97 (24.25%), and pyrexia, coughing, nasal discharge, and lacrimation combinedly found in 31 (7.75%), while all of the above signs were found in 10 (2.5%) goats. Similar signs were reported by many researchers [15, 34, 40, 46]. These findings are further supported by that of mycoplasma-infected goats showing high body temperature, painful respiration, and persistent cough [42]. It is justified that most of the *Mycoplasma* species present similar signs and symptoms.

Pathological lesions play an important role in the proper diagnosis of the disease. Pathological lesions provide evidence for pathologists to evaluate the severity of infection. The present study was carried out for the necropsy of a total of 200 goats across the southern areas (Khan, Bannu, Karak, and Kohat) of Khyber Pakhtunkhwa. The lung lesion was recorded in 38% of dead/slaughtered goats comprising accumulation of straw-color fluid in the pleural cavity called pleural fluid (36 (18%)), consolidation, red hepatization (40 (20%)), cranio-ventral pneumonia, and unilateral infected lungs (42 (21%)) recorded in the thoracic cavity. The similar lesions were recorded in [19, 20, 40, 47, 48]. The Mccp infection is restricted to the thoracic cavity, and this is why the lesions are mainly limited to lung tissues. The histopathological lesions were found different in different lungs tissue samples from various number of goats including pulmonary emphysema was recorded in lung tissues rom 33 dead goats, leucocytic infiltration in 72, atelectasis in 30, and thickening of interlobular septa were observed in lung tissues from 34 goats . These observations were closely related to the findings of many researchers [19, 40, 42, 48–51].

## 5. Conclusion

*Mycoplasma capricolum* subsp. *capripneumoniae* (Mccp) was confirmed in southern areas of Khyber Pakhtunkhwa Pakistan by PCR and cELISA. PCR detected Mccp in 5.5% of goats in southern areas of Khyber Pakhtunkhwa. The cELISA kit detected antibodies against Mccp in 17% of goat serum in southern areas of Khyber Pakhtunkhwa. The highest prevalence of Mccp was found in collected samples from Kohat by PCR. The highest seroprevalence of Mccp was found in serum samples collected from Kohat by ELISA.

## Figures and Tables

**Table 1:** Seroprevalence of *Mycoplasma capricolum* subsp. *capripneumonaie* by cELISA in southern areas of Khyber Pakhtunkhwa.

Area	No. of samples	Positive samples	Negative samples	Prevalence (%)
Dera Ismail Khan	50	7	43	14%
Bannu	50	9	41	18%
Karak	50	4	46	8%
Kohat	50	14	36	28%
Total	200	34	166	17%

**Table 2:** Statistical analysis of seroprevalence of *Mycoplasma capricolum* subsp. *capripneumonaie* by cELISA in southern areas of Khyber Pakhtunkhwa.

Area	ELISA-confirmed Mccp		Total	Chi-sq	*P* value
	Positive	Negative			
Khan	7	43	50	7.52	0.05
Bannu	9	41	50		
Karak	4	46	50		
Kohat	14	36	50		
Total	34	166	200		

Statistical analysis by *χ*2 showed significant association (*P* > 0.05) among four different districts.

**Table 3:** Molecular identification of *Mycoplasma capricolum* subsp. *capripneumoniae* by PCR from the different clinical samples of goats in southern areas of Khyber Pakhtunkhwa, Pakistan.

Area	Samples	No. of samples	PCR positive	PCR negative	PCR prevalence percentage
Dera Ismail Khan	Nasal swab	50	2	48	4%
	Pleural fluid	50	3	47	6%
	Tissue	50	3	47	6%
	Total	150	8	142	5.33%

Bannu	Nasal swab	50	2	48	4%
	Pleural fluid	50	5	45	10%
	Tissue	50	2	48	4%
	Total	150	9	141	6%

Karak	Nasal swab	50	1	49	2%
	Pleural fluid	50	3	47	6%
	Tissue	50	1	49	2%
	Total	150	5	145	3.33%

Kohat	Nasal swab	50	5	45	10%
	Pleural fluid	50	4	46	8%
	Tissue	50	2	48	6%
	Total	150	11	139	7.33%

Grand Total		600	33	567	5.5%

**Table 4:** Statistical analysis of molecular identification of *Mycoplasma capricolum* subsp. *capripneumoniae* by PCR from the different clinical samples of goats in southern areas of Khyber Pakhtunkhwa, Pakistan.

Area	PCR confirmed Mccp		Total	Chi-sq	*P* value
	Positive	Negative			
Nasal swab	10	190	200	2.51	0.28
Pleural fluid	15	185	200		
Tissue	8	192	200		
Total	33	567	600		

Statistical analysis by *χ*2 showed a nonsignificant association (*P* > 0.05) among three different types of samples.

**Table 5:** Percentage of clinical signs in naturally infected goats suffering from respiratory syndrome in southern areas of Khyber Pakhtunkhwa, Pakistan.

S. no.	Clinical findings	Khan (n = 100)	Bannu (n = 100)	Karak (n = 100)	Kohat (n = 100)	Total (n = 400)	Sign (%)
1	Pyrexia	56	51	43	57	207	51
2	Cough	57	55	31	60	203	50.75
3	Pneumonia	58	50	28	57	193	48.25
4	Nasal discharge	70	62	37	73	242	60.5
5	Lacrimation	20	17	23	32	92	23
6	Conjunctivitis	29	23	19	26	97	24.25
7	Arthritis	3	1	1	5	10	2.5
8	Pneumonia + nasal discharge + lacrimation	7	9	7	8	31	7.75
9	All signs	3	1	1	5	10	2.5

**Table 6:** Occurrence of gross pathological lesions in the thoracic cavity in naturally infected goats.

Areas	No. of samples	Gross pathology			
		Grossly normal	Straw color fluid/pleural fluid	Consolidated lungs/red hepatization	Cranio-ventral pneumonia/unilateral infected lungs
DI Khan	50	26	11	13	13
Bannu	50	34	9	7	9
Karak	50	41	4	5	5
Kohat	50	23	12	15	15

**Table 7:** Microscopic lesions in naturally infected goats suspected of *Mycoplasma capriculom* subsp. *capripneumoniae* across the southern areas of Khyber Pakhtunkwa, Pakistan.

Areas	No. of samples	Histopathology			
		Pulmonary emphysema	Leucocytic infiltration	Atelectasis	Thickening of interlobular septa
DI Khan	50	12	22	8	10
Bannu	50	6	16	6	6
Karak	50	4	9	5	5
Kohat	50	11	25	11	13

## Data Availability

The data presented in this study are deposited and made publicly available in an acceptable repository, prior to publication.
